# Factors Affecting Complementary Feeding of Infants. A Pilot Study Conducted after the Introduction of New Infant Feeding Guidelines in Poland

**DOI:** 10.3390/nu13010061

**Published:** 2020-12-28

**Authors:** Malgorzata Kostecka, Izabella Jackowska, Julianna Kostecka

**Affiliations:** 1 Faculty of Food Science and Biotechnology, University of Life Sciences in Lublin, Akademicka 15, 20-950 Lublin, Poland; izabella.jackowska@up.lublin.pl; 2 Faculty of Medicine, Medical University of Lublin, Chodźki 19, 20-093 Lublin, Poland; kostecka.julianna@gmail.com

**Keywords:** breastfeeding, complementary foods, complementary feeding, infants, nutrition of knowledge, gluten-containing foods

## Abstract

Adequate nutrition in the first year of life is the key prerequisite for a child’s healthy growth and development. The success of complementary feeding is influenced by various factors, including the family’s socioeconomic status, maternal age, place of residence and educational level, older children and duration of breastfeeding. Modified infant feeding guidelines were introduced in Poland in 2016. The aim of this study was to identify the factors that exert the greatest influence on infant feeding practices in the Polish population. A thorough understanding of maternal factors that are responsible for undesirable feeding practices is required to improve the mothers’ knowledge and to promote healthy feeding practices. This study was carried out in March–June 2018 and between November 2018 and March 2019 in the Region of Lublin in southeastern Poland. The mothers of children aged 9–14 months, who had introduced complementary foods, were invited to the study. A total of 441 mothers agreed to participate, and 289 of them fully and correctly completed the questionnaires. Logistic regression models were developed to assess the association between maternal factors, such as age, educational level and the nutrition knowledge score, and introduction of solid foods. The infant’s sex and birth weight and the mother’s place of residence had no significant influence on the duration of breastfeeding. The mother’s age and educational level, the number of children in the family and maternal nutrition knowledge scores contributed to significant differences in breastfeeding duration. Older mothers (30–34 years vs. 25–29 years, *p* = 0.001), better educated mothers (university vs. secondary school education *p* = 0.002) and mothers with one or two children exclusively breastfed their children for longer (17 weeks vs. 11 weeks, *p* = 0.002) than younger mothers with secondary school education and more than three children. Exclusive formula feeding was significantly correlated with untimely introduction of solid foods compared to exclusive breastfeeding (13 weeks vs. 19.7 weeks, *p* = 0.001). Mothers with nutrition knowledge scores in the upper tertile were more likely to adapt food consistency to the skills manifested by the child (88%) (OR = 1.88; Cl: 1.53–2.26, *p* < 0.05) and were less likely to delay the introduction of new foods that required chewing and biting (84%) (OR = 0.22; Cl: 0.09–0.34, *p* < 0.05) than mothers with nutrition knowledge scores in the bottom tertile. Maternal age, educational level and nutrition knowledge significantly increased the age at which infants were introduced to solid foods and the correct identification of the signs indicative of the child’s readiness to explore new tastes and foods with a new consistency. The above factors contributed to delayed, but not untimely, introduction of complementary foods (6 months of age or later), including gluten.

## 1. Introduction

Adequate nutrition in the first year of life is the key prerequisite for a child’s healthy growth and development. Exclusive breastfeeding implies that the infant only receives breast milk, whereas exclusive formula feeding implies that the child has been exclusively fed a milk replacement formula selected by the parents or the pediatrician since birth. The timing of the introduction of solid food during infancy may have effects on life-long health [1]. Building on prior recommendations for the timing of the introduction of solids [2], in its 2005 policy statement, the American Academy of Pediatrics (AAP) Section on Breastfeeding encouraged delaying the introduction of solid foods (including cereals) until the age of six months for exclusively breastfed infants and until the age of four months for formula-fed infants [3]. Notably, with the 2012 revision of this recommendation by the AAP, the recommended age for the introduction of solid foods was increased from four to six months [4]. The WHO recommends that infants should be introduced to complementary foods at six months of age [5], and similar guidelines have been formulated by ESPGHAN, NASPGHAN and the European Academy of Allergy and Clinical Immunology (EAACI), which recommend that complementary foods be introduced between weeks 17 and 26 [6].

Modified and expanded infant feeding guidelines were introduced in Poland in 2016 [7]. These guidelines have been proposed by the Division of Nutrition of the Polish Society for Pediatric Gastroenterology, Hepatology and Nutrition with the aim of adapting the recommendations formulated by scientific societies and expert teams to Polish conditions [6,8,9,10]. The formulated recommendations and guidelines define:

- estimated portion size and the daily number of meals that should be consumed by children from different age groups,
- eating habits developed by children in different age groups, and
- type, consistency and order in which different foods should be introduced to a child’s diet.

Polish infant feeding guidelines are presented in a diagram covering the first 12 months of a child’s life (number of meals, portion size, child’s skills, and sample meals). According to recommendations, complementary foods should be introduced between the ages of 17 and 26 weeks, which is when most infants develop the ability to ingest solid foods. At this age, children learn to maintain a supported sitting posture, they develop neuromotor skills required for controlling head and neck movement and eating from a spoon. Motor coordination (hand-mouth) and the ability to chew allow for the safe introduction of new foods. The tongue-thrust reflex that prevents newborns and infants from choking on foreign objects and disables the administration of non-liquid foods disappear during this period [7].

Complementary feeding is defined as the process starting when breast milk or formula fed alone are no longer sufficient to meet the nutritional requirements of infants, and therefore other foods and liquids are needed, along, with breast milk or formula. Complementary food (CF) is defined as any food, solid or (semi) liquid, besides breast milk or its substitutes, i.e., infant (or follow-on) formula. This definition of complementary food was chosen because not all infants are breastfed or are only breastfed for short periods [11]. Complementary foods are an important stage in the transition from milk feeding to family foods. The period during which complementary foods are introduced to the infant’s diet is marked by rapid growth and development. During this period, infants are susceptible to nutrient deficiencies and excesses, and marked dietary changes occur as children become exposed to new foods, tastes, and feeding experiences [12].

Complementary feeding (CF) introduced before four months of age can lead to diarrhea, colic, abdominal pain and higher risk of overweight and obesity in later stages of life [13]. However, delayed introduction of solid foods can contribute to developmental disorders, malnutrition (dietary iron deficiency beyond the age of six months), eating disorders (delayed introduction of solid foods that stimulate healthy development of oral muscles, biting and chewing abilities), overweight and obesity [14,15]. The success of complementary feeding is influenced by various factors, including the family’s socioeconomic status, maternal age, place of residence and educational level, older children and duration of breastfeeding.

The aim of this study was to identify the factors that exert the greatest influence on infant feeding practices in the Polish population. A thorough understanding of maternal factors that are responsible for undesirable feeding practices is required to improve the mothers’ knowledge and promote healthy feeding practices.

## 2. Methods

### 2.1. Study Design and Participants

This study was carried out in March–June 2018 and between November 2018 and March 2019 in the Region of Lublin in southeastern Poland. The mothers of children aged 9–14 months who were patients of several pediatrics clinics in Lublin and in towns of Lublin county were invited to participate. A total of 441 mothers consented to participate in the study. The inclusion criterion was a healthy child without metabolic disorders or other disorders that require an elimination diet or foods for special medical purposes. Breastfeeding was not a requirement for participation. The exclusion criteria were: prematurity and low birth weight requiring nutritional support (enteral or parenteral nutrition) in the first weeks of a child’s life. Mothers self-completed the questionnaire. The completed questionnaires were verified for completeness to ensure that they met the required quality standards.

### 2.2. Data Collection

Out of the 441 returned questionnaires, 289 fully and correctly completed questionnaires were qualified for the study. One hundred and fifty-two questionnaires were eliminated from further analysis and destroyed due to incorrect marking, absent responses to some questions or failure to complete the knowledge test in the second part of the study. The research tool was an original questionnaire developed by the authors of the study based on the Infant and Young Child Feeding (IYCF) assessment [16] and the Complementary Food Frequency Questionnaire [17,18]. Selected questions from validated questionnaires were translated into Polish and modified to include foods that are commonly present in the diet of Polish infants and small children.

In the first part of the study, mothers filled in a questionnaire (in paper form) containing 49 questions on breastfeeding (duration and frequency), milk replacement formulas (breast milk replacement), introduction of solid foods, factors that determine the introduction of complementary feeding, types of solid foods and frequency of complementary meals, cooking techniques, infant behaviors during feeding and symptoms of the child’s readiness for solid foods (for example: sitting position, ability to chew, interest in new products, and motor coordination). In the second part, the mothers were asked several short questions (eight questions assessed maternal nutrition knowledge and familiarity with Polish infant feeding guidelines: the recommended duration of breastfeeding, development of feeding skills, portion size, solid foods that should be introduced first in complementary feeding, changes in meal consistency, mother’s role, children’s role), and their knowledge was verified by a researcher/nurse/pediatrician. The mothers wrote their responses in the paper questionnaire.

In the light of Polish regulations on questionnaire surveys, the study did not require the consent of a bioethics committee, only the informed consent of the mothers who volunteered to fill in the questionnaires.

### 2.3. Statistical Analysis

Categorical variables were presented as the percentage of samples (%), and continuous variables were presented as the median and the interquartile range (IQR). The differences between groups were analyzed with a chi-square test (categorical variables) or a Mann-Whitney test (continuous variables). Before statistical analysis, the normality of the distribution of the variables was checked with a Kolmogorov-Smirnov test.

The odds ratios (ORs) and 95% confidence intervals (95% CIs) were calculated. The outcome represented the mothers’ compliance with feeding guidelines, including the introduction of solid foods between the age of four to six months, and the introduction of an adequate number of complementary meals based on the child’s age. The ORs were adjusted for maternal age (years), maternal nutrition knowledge score (points), maternal educational level, feeding method at three months of age, and having at least one child. The significance of ORs was assessed by Wald’s statistics. For all tests, *p* < 0.05 was considered as significant. Analyses were performed using Statistica software (version 13.1 PL; StatSoft Inc., Tulsa, OK, USA; StatSoft, Krakow, Poland).

## 3. Results

The mothers differed in age, number of children, maternal nutrition knowledge score, place of residence and educational level (Table 1).

In the studied population, 61% of the mothers exclusively breastfed three-month-old infants, 21% combined breast milk with formulas, and 18% fed formulas only. At six months of age, the percentage of exclusively breastfeeding mothers decreased to 38.8%, and the percentage of mothers who fed modified milk only increased to 34.25%. At nine months of age, the percentage of exclusively breastfeeding mothers decreased to 21% (in addition to receiving solid food, children were breastfed and not formula fed), and the percentage of mothers who fed modified milk only increased to 46%. Mothers aged 30–34 years were significantly more likely (*p* < 0.05) to continue breastfeeding compared to mothers aged 25–29 were significantly more likely (*p* < 0.05) to continue formula feeding.

### 3.1. Factors Associated with the Time of Introduction of Complementary Foods

Older mothers (>35 years vs. 25–29 years, *p* = 0.001), better educated mothers (university vs. secondary school education, *p* = 0.002) and mothers of one or two children who exclusively breastfed their children for longer periods of time (17 weeks vs. 11 weeks, *p* = 0.002) than younger mothers with secondary school education and more than three children. The mother’s place of residence had no significant influence on the duration of breastfeeding.

An analysis of the research results revealed that complementary foods were introduced at the average age of 17 weeks. Vegetables, mostly carrot puree, were the first solid foods to be introduced to infant diets (83.0%), followed by apple puree, gluten-free pudding, juice and soup. The influence of environmental and maternal factors on the time of introduction of complementary foods was analyzed (Table 2), and the results were compared against the guidelines: according to Polish infant feeding guidelines, solid foods should be introduced by the fourth month, but not later than the sixth month of life (between 16 and 27 weeks of age). Exclusive formula feeding was significantly correlated with the untimely introduction of solid foods (OR = 2.01; 95% CI: 1.54–2.24; *p* < 0.0001), compared to the reference group (exclusive breastfeeding). Low maternal education significantly accelerated the introduction of solid food before the recommended date compared to mothers with university education (OR =1.79; Cl: 1.23–1.97, *p* < 0.001). The mothers of older children introduced complementary foods significantly earlier (before 4 months) (OR = 1.51; Cl: 1.19–1.77, *p* < 0.001) than first-time mothers who introduced solid foods significantly later, often after the recommended age of 26 weeks (OR = 1.84; Cl: 1.36–2.20; *p* < 0.001). Nutrition knowledge scores in the upper tertile and the bottom tertile influenced the introduction of complementary feeding. Mothers with a nutrition knowledge score in the bottom tertile (54%) were more likely to introduce solid foods prematurely (OR = 1.54; Cl: 1.26–1.79, *p* < 0.01), whereas mothers with a score in the upper tertile (72%) were significantly less likely to introduce solid foods before four months (OR = 1.72; Cl: 1.46–1.94, *p* < 0.001).

### 3.2. Complementary Feeding (CF)

All maternal factors significantly affected complementary feeding and the age at which complementary foods were introduced. According to most respondents, vegetables and fruit should be introduced to infant diets at six months, but many mothers introduced these products earlier. Maternal age influenced the age at which gluten-containing foods were introduced (Table 3). Mothers older than 35 years introduced gluten between four and six months of age, and we are of the opinion that both the time of introduction and the amount of gluten in the daily diet were important (*p* = 0.0002). Food allergies did not affect the age at which gluten-containing foods were introduced (*p* = 0.231), but food allergies in the family significantly delayed gluten introduction up to the age of eight months (*p* = 0.0001). The presence of older children in the family also significantly delayed gluten introduction (*p* = 0.002).

An analysis of the participants’ responses revealed that eggs and fish were introduced to infant diets significantly past the recommended dates, usually after eight months of age, regardless of maternal age, educational level and nutrition knowledge score. Cow’s milk was incorporated into infant diets prematurely, and mothers with secondary education and more than one child introduced cow’s milk significantly earlier (before eight months of age) (*p* = 0.001) compared to mothers with university education and one child only, most of whom introduced cow’s milk past the age of 12 months (*p* = 0.003). According to Polish guidelines, cow’s milk should not be incorporated into infant diets earlier than 12 months of age, in a daily amount of up to 500 mL.

Children with a food allergy and children from families with a history of food allergies received cow’s milk significantly later, beyond the age of 12 months (*p* = 0.001). Mothers with nutrition knowledge scores in the bottom tertile (53%) were significantly more likely to introduce cow’s milk and dairy products prematurely (OR = 1.53; Cl: 1.17–1.84, *p* < 0.05), whereas mothers with scores in the upper tertile (65%) more often introduced cow’s milk beyond the age of 12 months (OR = 1.65; Cl: 1.38–1.87, *p* < 0.05). More than 1/5 of the respondents prematurely introduced their children to honey, mushrooms and processed meat, and the predisposing factors were secondary education, more than one child in the family and nutrition knowledge scores in the bottom tertile (*p* < 0.05). According to the American Academy of Pediatrics, the National Honey Board, and the Polish Society for Pediatric Gastroenterology, Hepatology and Nutrition, honey should not be administered before 12 months of age due to the risk of infant botulism.

### 3.3. Number of Complementary Meals

In line with the current infant feeding guidelines, the number of meals per day should be adapted to the child’s age (Table 4). According to Polish guidelines, breast milk and milk formulas should be the mainstays of infant feeding between 6 and 12 months of age. Two complementary meals are recommended between 6 and 8 months, and four complementary meals should be served in the following months. Having at least one older child was a factor that significantly differentiated the number of complementary meals fed to infants between 6 and 8 months of age (*p* < 0.05) and >12 months of age (*p* < 0.05). Mothers of more than one child served a higher than the recommended number of complementary meals. Maternal nutrition knowledge scores also differentiated the number of complementary meals. Mothers with scores in the bottom tertile were more likely to serve too many meals between 6 and 8 months of age (71%) and too few complementary meals between 9 and 11 months of age (56%). Mothers with scores in the upper tertile (84%) more often served the recommended number of complementary meals.

### 3.4. Feeding Behavior of Infants

The maternal nutrition knowledge score (points) and maternal age differentiated the respondents’ assessment of the child’s readiness for solid foods. Older mothers (>35 years vs. 19–24 years, *p* < 0.05) were more likely to recognize symptoms of readiness such as sitting position, reaching for food, ability to hold a training cup without assistance, ability to chew, interest in new products and foods. Polish infant feeding guidelines list child development abilities by age, such as the ability to eat off the spoon at 7–8 months of age. Mothers with nutrition knowledge scores in the upper tertile were more likely to adapt food consistency to the skills manifested by the child (88%) (OR = 1.88; Cl: 1.53–2.26, p < 0.05) and were less likely to delay the introduction new foods that required chewing and biting (84%) (OR = 0.22; Cl: 0.09–0.34, p < 0.05).

## 4. Discussion

In the studied group, only 30.8% of the mothers were familiar with the WHO recommendations and the guidelines developed by the Division of Nutrition of the Polish Society for Pediatric Gastroenterology, Hepatology and Nutrition regarding the duration of breastfeeding were more likely to apply these recommendations in practice. Similar results were reported by Cresswell [19] and Senghore [20] in the African population and by Stough [21] in the US population. In turn, Owais [22] observed that 19% of mothers were familiar WHO recommendations regarding the age at which complementary feeding should be initiated. Early introduction of complementary foods (i.e., before the infant reaches six months of age) may have the effect of replacing breast milk and halting breastfeeding altogether at too early a stage [23,24]. Many children younger than six months are not yet physiologically ready to receive complementary food because their nervous system, gastrointestinal tract and kidneys are still underdeveloped [23].

Maternal educational level has a direct positive impact on the linear growth of infants [25,26]. Mothers with high levels of nutrition knowledge have been shown to modify infant diets based on the child’s abilities and rely on a critical window of opportunity to introduce new products and foods with a new consistency [27,28]. In the studied population, the factors that motivated mothers to introduce solid foods prematurely were the child’s appetite for new products and insufficient (in the mothers’ opinion) milk intake. Older mothers (30–40 years and older) and mothers with nutrition knowledge scores in the bottom tertile were significantly more likely to begin complementary feeding earlier, as soon as the child demonstrated an interest in table foods (*p* = 0.0031). According to a review article by Wijndaele et al., younger mothers are also likely to begin complementary feeding earlier when they observe signs of readiness in their children [29]. According to research [30], there are two main reasons why complementary foods are introduced before 17 weeks of age were: the baby is hungry and, in the mother’s opinion, the child is old enough to be given solid foods. Similar findings were reported by Scott et al. in the Perth Infant Feeding Study [13], Clayton in the Infant Feeding Practices Study II [4] and Gross in an urban Latina WIC population [31]. Similarly to our study, Clayton [4] also found that exclusive formula feeding significantly contributed to early introduction of solid foods to infant diets. Untimely introduction of complementary feeding is a global problem, particularly in Latin America, the Caribbean, and East Asia Pacific, where almost half of the children receive CF between four and five months of age [32].

An analysis of the factors that influence maternal decisions on the early introduction of solid foods provides very important insights. In many European [33,34] and American studies [35], the main factor was low educational level, and to a lesser degree, the mother’s age. In a Polish study by Zielinska, the odds of being introduced to complementary foods early (<4 months) were higher among children of less well educated mothers. Research conducted in Austria revealed higher odds among children of less well educated mothers aged 25–29 years [36]. However, some studies found that educational level was inversely associated with the age of complementary feeding only in mothers of Western or Caucasian origin [37,38].

According to Polish and ESPGHAN guidelines, gluten should be incorporated into infant diets on the same principles as other complementary foods, i.e., between four and six months of age, to reduce the likelihood of developing wheat allergy [6]. In the studied population, mothers with older children were more likely to delay the introduction of gluten. This trend is consistent with older guidelines for the prevention of celiac disease, which stipulate that cereals and gluten-containing products should be introduced only around one year of age. According to the latest knowledge, delayed introduction of gluten beyond seven months does not have a protecting effect and could even increase the risk of celiac disease by 25% [39,40].

In our study, mothers generally introduced potentially allergenic solid foods later than recommended. Eggs, fish and nuts were usually incorporated into infant diets between 8 and 12 months of age regardless of genetic predisposition to allergy (*p* = 0.239). In our study, the introduction of potentially allergenic solid foods was delayed due to the risk of serious allergic reactions, including asthma and anaphylactic shock. According to the recommendations for infants with a low risk of developing food allergies, potentially allergenic foods should be introduced at the same age as other solid foods, i.e., between four and six months of age when children show an interest in eating solids, and they should be served at least three times per week [41]. The risk of food allergy is minimized when eggs and peanuts are introduced between four and six months of age [42,43,44]. According to Polish guidelines, whole eggs should be introduced together with other complementary foods (three–four times per week). Oily sea fish (herring, salmon, sprat, halibut, and cod) should be administered once a week, interchangeably with meat, starting at six months [7]. In the LEAP study, early introduction of small quantities of nuts significantly decreased the risk of allergy in later life, including in the high-risk group, relative to children who were introduced to nuts beyond the age of 1 year (3.2% vs. 13.2%, *p* < 0.001) [45]. The National Institute of Allergy and Infectious Diseases recommends early peanut introduction, and according to the addendum guidelines, peanuts should be introduced as early as at four-six months of age in high-risk infants with severe eczema and/or egg allergy [46].

Complementary feeding is also influenced by the infant’s readiness to eat solid foods with different consistency. In this study, the vast majority of the mothers (93.4%) were of the opinion that unassisted sitting or sitting with leaning on the arms were the key symptoms manifesting a child’s ability to eat solid foods. Reaching for food was an important sign for 2/3 of the respondents, whereas the ability to chew was recognized as a crucial symptom by every third mother. According to the American Academy of Pediatrics (AAP), semi-solid foods are a significant change and should not be introduced until six months of age. This age usually coincides with the neuromuscular development necessary to eat solid foods [47]. Early introduction of lumpy foods (before 10 months) is associated with a more varied diet, including greater variety of fruit and vegetables, at seven years of age [48]. Many parents make the mistake of delaying the introduction of pureed and lumpy foods [49].

In the surveyed group, vegetables were the first solid foods to be introduced to infants (83.0%). This is consistent with nutritional recommendations, which state that complementary feeding should begin with low energy and/or low protein-dense foods such as vegetables [50,51]. Longitudinal data from the Avon Longitudinal Study of Parents and Children (ALSPAC) indicate that exposure to home prepared fruit and vegetables during the early stages of complementary feeding is positively associated with the frequency and variety of intake of these foods at seven years of age [52,53]. The introduction of vegetables before other foods is an effective approach to expanding a child’s diet, promoting an interest in tastes other than the sweet taste, and gradually introducing the child to different sweet-tasting foods. Some authors have argued that introducing fruit before vegetables reinforces infants’ innate preference for sweet foods and discourages the acceptance of savory foods, such as vegetables [54]. In the present study and in the research conducted by Barends et al. [55], fruit was more readily accepted than vegetables from the start, which indicates that the introduction of vegetables as the first solid foods leads to a more varied diet and promotes the acceptance of new tastes. At 12 months of age, daily vegetable intake in infants who had been introduced exclusively to vegetables for the first 2 weeks of complementary feeding was 38% higher than in those who had been introduced to fruit first [55].

The cessation of exclusive breastfeeding is the main problem in early complementary feeding. According to a Polish report on breastfeeding, mothers cease to exclusively breastfeed and introduce complementary foods on the assumption that the child is ready to try new tastes and that breast milk alone fails to meet the child’s energy needs [56]. Despite the absence of convincing evidence that the early introduction of solid foods (before six months of age) has negative health implications, shorter duration of exclusive breastfeeding minimizes the health benefits for both the mother and the child [2,57].

### Strengths and Limitations

The greatest strengths of this study were a large sample size and the children’s age (9–14 months), which provided the collection of accurate and relatively recent information on complementary feeding and breastfeeding in the first six months of life. The conducted survey supported an evaluation of maternal nutrition knowledge which was compared with daily practices regarding complementary feeding, the time of introduction of complementary foods and the number of complementary meals. The study also provided valuable information about breastfeeding and formula feeding at three, six and nine months of age. The factors that influence the age of complementary feeding have been insufficiently investigated in the Polish population. The present study describes Polish mothers’ compliance with the new infant feeding guidelines.

The main weakness of the study was the high percentage of incorrectly filled questionnaires that were rejected as well as the absence of fathers. The fathers’ nutrition knowledge and their influence on the children’s eating habits could not be evaluated. In the future, the survey could be repeated with the involvement of both parents to compare the mothers’ and the fathers’ nutrition knowledge and their ability to apply that knowledge to practice.

## 5. Conclusions

This study demonstrated that maternal age, educational level and nutrition knowledge significantly influenced the duration of breastfeeding, the age at which infants were introduced to solid foods and the correct identification of the signs indicative of the child’s readiness to explore new tastes and foods with a new consistency. Factors that prolong breastfeeding and influence the timely introduction of complementary solid foods were identified. Despite the fact that Polish infant feed guidelines had been developed in the form of a simple diagram, they were not comprehensible for all mothers and mothers with older children were more likely to disregard the new guidelines and feed infants based on previous experiences. Infant feeding charts were not always sufficient for young mothers and mothers with low educational attainment who should receive additional assistance. The results of this study can be used by pediatricians and dietitians to educate mothers about infant nutrition. The described maternal factors can be analyzed by health professionals to identify mothers who are least likely to follow nutritional recommendations.

## Figures and Tables

**Table 1:** Maternal factors.

Maternal Factors	Number (%) 289 (100)	*p*-Value
Age of mothers (years, average), mean (95% Cl)		0.02
19–24–21.4 on average (19.8; 23.7)	97 (33.6)	
25–29–26.1 on average (25.4; 29.6)	41 (14.2)	
30–34–31.3 on average (30.1; 33.2)	60 (20.8)	
>35–36.9 on average (35.2; 38.4)	91 (31.4)	
Place of residence, *n* (%)		0.01
Rural	71 (24.6)	
Urban	218 (75.4)	
Educational level, *n* (%)		0.04
University	245 (84.7)	
Secondary school	39 (13.6)	
Primary school	5 (1.7)	
Age of solid food introduction (weeks)		0.002
<17	97 (33.6)	
17–26	181 (62.6)	
>26	11 (3.8)	
Number of children		0.005
1	123 (42.6)	
2	131 (45.3)	
>3	35 (12.1)	
Maternal nutrition knowledge score (points),		0.03
Low (0–4 points)	72 (24.9)	
Medium (5–6 points)	171 (59.2)	
High (7–8 points)	46 (15.9)	

**Table 2:** Odds ratios (95% confidence interval). The associations between maternal factors, feeding method in the 3rd month of life and age at which solid foods were introduced.

	Introduction of Solid Foods before 4 Months of Age (Ref.: Introduction of Solid Foods between 4 and 6 Months of Age)	Introduction of Solid Foods after 6 Months of Age (Ref.: Introduction of Solid Foods between 4 and 6 Months of Age)
Maternal age		
Older mothers (ref. mothers <35 years)	0.25 *** (0.13; 0.41)	1.97 **** (1.65; 2.21)
Maternal educational level		
Secondary school (ref. University)	1.79 *** (1.23; 1.97)	0.93 (0.74; 1.12)
Feeding method in the 3rd month of life		
Fully formula fed (ref. Exclusive breastfeeding)	2.01 **** (1.54; 2.24)	0.23 *** (0.11; 0.36)
Maternal nutrition knowledge score (points)		
Low (ref. High)	1.54 ** (1.26; 1.79)	0.52 ** (0.27; 0.69)
Having at least one older child		
Yes (ref. no older children)	1.51 ** (1.19; 1.77)	1.22 (0.94; 1.43)

Statistically significant: ** *p* < 0.01; *** *p* < 0.001; **** *p* < 0.0001, ns- not significant.

**Table 3:** Odds ratios (95% confidence interval). The associations between maternal factors and the introduction of gluten, cow’s milk and honey according to Polish guidelines.

	Introduction of Cow’s Milk before 12 Months of Age (Ref.: after 12 Months of Age)	Introduction of Gluten-Containing Foods before 4 Months of Age (Ref.: between 4 and 6 Months of Age)	Introduction of Nuts after 6 Months of Age (Ref.: between 4 and 6 Months of Age)	Introduction of Honey before 12 Months of Age (Ref.: after 12 Months of Age)
Maternal age				
Older mothers (ref. mothers <35 years)	1.08 (0.89–1.26)	0.77 * (0.56–0.98)	1.12 (0.88–1.31)	0.53 ** (0.34–0.84)
Maternal nutrition knowledge score (points)				
Low (ref. High)	1.97 ** (1.45–2.16)	1.21 * (1.03–1.68)	1.46 * (1.23–1.74)	1.46 * (1.13–1.79)
Having at least one older child				
Yes (ref. no older children)	1.31 * (1.06–1.43)	1.06 (0.88–1.14)	1.19 (1.03–1.41)	1.11 (0.95–1.19)
Maternal educational level				
Secondary school (ref. university)	1.59 * (1.23–1.85)	1.36 * (0.94–1.49)	1.39 * (1.24–1.59)	1.56 * (1.21–1.87)

Maternal nutrition knowledge score: low (0–4 points), high (7–8 points). Statistical significance: * *p* < 0.05; ** *p* < 0.01.

**Table 4:** Odds ratios (95% confidence interval). The associations between maternal factors and the number of complementary meals.

	More Than 2 Complementary Meals between 6 and 8 Months of Age (Ref.: 2 Complementary Meals)	More Than 3 Complementary Meals between 9 and 11 Months of Age (Ref.: 3 Complementary Meals)	More Than 4 Complementary Meals at >12 Months of Age (Ref.: 4 Complementary Meals)
Maternal nutrition knowledge score (points)			
Low (ref. High)	1.94 ** (1.56–2.24)	0.79 * (1.33–2.08)	1.09 (0.95–1.19)
Having at least one older child			
Yes (ref. no older children)	1.29 * (1.06–1.43)	1.11 (0.97–1.19)	2.08 *** (1.64–2.39)
Maternal educational level			
Secondary school (ref. university)	1.46 * (1.29–1.69)	1.09 (0.89–1.12)	1.99 *** (1.74–2.16)

Maternal nutrition knowledge score: low (0–4 points), high (7–8 points), Statistically significant: * *p* < 0.05; ** *p* < 0.01; *** *p* < 0.001.

## Data Availability

The data that support the findings of this study are available from **M**algorzata Kostecka (MK) University of Life Science, but restricted for research use only. The data are not publicly available. Data are available from the authors upon reasonable request and with permission of University of Life Science.
